# Association of Kidney Disease Measures with Cause-Specific Mortality: The Korean Heart Study

**DOI:** 10.1371/journal.pone.0153429

**Published:** 2016-04-19

**Authors:** Yejin Mok, Kunihiro Matsushita, Yingying Sang, Shoshana H. Ballew, Morgan Grams, Sang Yop Shin, Sun Ha Jee, Josef Coresh

**Affiliations:** 1 Department of Epidemiology, Bloomberg School of Public Health, Johns Hopkins University, Baltimore, MD, United States of America; 2 Department of Epidemiology and Health Promotion, and Institute for Health Promotion, Graduate School of Public Health, Yonsei University, Seoul, Korea; 3 Department of Internal medicine, Korea Medical Institute, Seoul, Korea; Mario Negri Institute for Pharmacological Research and Azienda Ospedaliera Ospedali Riuniti di Bergamo, ITALY

## Abstract

**Background:**

The link of low estimated glomerular filtration rate (eGFR) and high proteinuria to cardiovascular disease (CVD) mortality is well known. However, its link to mortality due to other causes is less clear.

**Methods:**

We studied 367,932 adults (20–93 years old) in the Korean Heart Study (baseline between 1996–2004 and follow-up until 2011) and assessed the associations of creatinine-based eGFR and dipstick proteinuria with mortality due to CVD (1,608 cases), cancer (4,035 cases), and other (non-CVD/non-cancer) causes (3,152 cases) after adjusting for potential confounders.

**Results:**

Although cancer was overall the most common cause of mortality, in participants with chronic kidney disease (CKD), non-CVD/non-cancer mortality accounted for approximately half of cause of death (47.0%for eGFR <60 ml/min/1.73m^2^ and 54.3% for proteinuria ≥1+). Lower eGFR (<60 vs. ≥60 ml/min/1.73m^2^) was significantly associated with mortality due to CVD (adjusted hazard ratio 1.49 [95% CI, 1.24–1.78]) and non-CVD/non-cancer causes (1.78 [1.54–2.05]). The risk of cancer mortality only reached significance at eGFR <45 ml/min/1.73m^2^ when eGFR 45–59 ml/min/1.73m^2^ was set as a reference (1.62 [1.10–2.39]). High proteinuria (dipstick ≥1+ vs. negative/trace) was consistently associated with mortality due to CVD (1.93 [1.66–2.25]), cancer (1.49 [1.32–1.68]), and other causes (2.19 [1.96–2.45]). Examining finer mortality causes, low eGFR and high proteinuria were commonly associated with mortality due to coronary heart disease, any infectious disease, diabetes, and renal failure. In addition, proteinuria was also related to death from stroke, cancers of stomach, liver, pancreas, and lung, myeloma, pneumonia, and viral hepatitis.

**Conclusion:**

Low eGFR was associated with CVD and non-CVD/non-cancer mortality, whereas higher proteinuria was consistently related to mortality due to CVD, cancer, and other causes. These findings suggest the need for multidisciplinary prevention and management strategies in individuals with CKD, particularly when proteinuria is present.

## Introduction

Chronic kidney disease (CKD), defined as reduced kidney function and/or kidney damage, is a worldwide public health problem [1–3]. The prevalence of CKD is 10–15% in Asia, Europe, and the USA [1, 3–6]. Cardiovascular disease (CVD) is an important complication of CKD since up to half of this population dies from CVD [7–14]. Indeed, numerous studies have reported the significant associations of lower estimated glomerular filtration rate (eGFR) and higher proteinuria with CVD mortality [10, 13–15].

Several studies also report the associations of these CKD measures with all-cause mortality [7–11]. This may merely reflect the link between CKD and CVD mortality, given that CVD is a leading cause of death in many places of the world [16]. On the other hand, reduced kidney function may relate to mortality due to cancer, the other worldwide leading cause of death [17–19]. However, to our knowledge, the only study to simultaneously investigate both key CKD measures, eGFR and proteinuria [20], did not find a significant association between CKD and cancer mortality [12]. Thus, further studies are needed, particularly to investigate the relationship between proteinuria and cancer mortality.

Although CVD and cancer are clearly important as causes of death, other causes account for approximately 30–35% of deaths in developed countries [16], and thus also warrant attention. A few studies have investigated the association of CKD with deaths due to non-CVD/non-cancer causes [17, 19, 21, 22]. Infection-related mortality has been most extensively examined in this context but has demonstrated conflicting results across studies [17, 19, 21, 23]. Moreover, of these, only one study explored proteinuria [21], and mortality causes other than infectious disease have not been systematically investigated [17, 19, 21–23].

Since prevention and management can be substantially different across diseases, simultaneously quantifying the respective associations of CKD measures with cause-specific mortality in a single cohort is important from both clinical and public health perspectives. Therefore, we assessed whether creatinine-based eGFR and dipstick proteinuria were associated with mortality due to CVD, cancer, and other causes in more than 350,000 Korean adults. The large sample size allowed us to also investigate finer representative causes of death.

## Materials and Methods

### Study population

The Korean Heart Study (KHS) is a retrospective cohort study based on data from private health examinations conducted at 18 centers in South Korea from 1996–2004 [24]. Of these centers, 14 centers opted in to the present cause-specific mortality study. The record linkage for mortality was based on an official personal identification number and identified 521,585 study members aged 20 years or older with a health assessment at baseline. We excluded participants with missing information on serum creatinine (n = 37,196) and dipstick urinary test (n = 116,457), leaving the final study population of 367,932 participants. The Institutional Review Board of Human Research of Yonsei University and all the individual centers participating in the KHS approved the investigation.

### Data collection

Demographic information, smoking status (never, past, or current), and medical history of comorbidities including CVD and cancer were assessed with questionnaires. Weight and height measurements were recorded while participants were wearing light clothing. Trained staff measured blood pressure. Blood samples were obtained after 12h of fasting. Total cholesterol, triglyceride, high-density lipoprotein cholesterol, fasting glucose, and liver enzymes (aspartate aminotransferase and alanine aminotransferase) were measured at each center using auto analyzers. The seropositivity to hepatitis B and C viruses was measured with radioimmunoassay or reverse passive haemagglutination in participating laboratories. Each center had internal and external quality control procedures as required by the Korean Association of Laboratory Quality Control [24]. Diabetes was defined as fasting glucose ≥126 mg/dl, use of glucose-lowering medication, or history of diabetes.

### Assessment of eGFR and dipstick proteinuria

Serum creatinine was measured using the kinetic rate Jaffe method, and GFR was estimated by the CKD-EPI equation, after reducing the creatinine level by 5%, the calibration factor to adjust a nonstandardized assay to creatinine standardized to isotope dilution mass spectrometry at the Cleveland Clinic Laboratory [25, 26]. Proteinuria was assessed by dipstick test using an automated urine analyzer, and was recorded as negative, trace, 1+, 2+, 3+, and 4+ in all centers except one systematically recording negative and trace together as low proteinuria.

### Follow-up for mortality

Death certificate linkage data until 31 December 2011 were provided by the Korean National Statistical Office. Based on the ICD code for primary cause of death, mortality was categorized into CVD (Disease of circulatory system: ICD-10 codes I00-I99 and sudden death: R96), cancer (C00-C97), and other (non-CVD/non-cancer) causes (all other ICD-10 codes) (S1 Table).

### Statistical analysis

Baseline characteristics are presented as mean and standard deviation (SD) for continuous variables and percentage for categorical variables across categories of eGFR (≥90, 60–89, 45–59, and <45 ml/min/1.73m^2^) and dipstick proteinuria (none/trace, 1+, 2+, and ≥3+). We combined all participants with eGFR <45 ml/min/1.73m^2^ since few participants (n = 168 [0.05%]) had eGFR <30 ml/min/1.73m^2^. The age-standardized cause-specific mortality was calculated across these categories using the Korean Population and Housing Census data in 2010 as a standard population [27].

Subsequently, we quantified the associations of eGFR and proteinuria with cause-specific mortality using Cox proportional hazards models. Since a J-shaped association has been seen for eGFR and morality risk [20, 28] and the lowest risk category varies across studies, for this analysis [8, 10, 14], we subdivided ≥90 and 60–89 ml/min/1.73m^2^ into ≥105, 90–104, 75–89, and 60–74. eGFR 75–89 ml/min/1.73m^2^ was the most prevalent category in those aged ≥50 years, a group with higher mortality than younger individuals, and thus we selected this category as the reference, as done previously [12, 29]. For dipstick proteinuria, we selected none/trace as the reference since one center recorded negative and trace proteinuria as a single category. However, using data from the other 13 centers, we also tested dipstick negative as a reference. Three models were constructed to evaluate the impact of potential confounders. Model 1 was unadjusted, and Model 2 incorporated age and gender. Model 3 further adjusted for smoking, body mass index, total cholesterol, systolic blood pressure, antihypertensive medications, diabetes, and history of CVD and cancer, and each of the CKD measures, as appropriate (proteinuria was incorporated in the analyses for eGFR and vice versa). We implemented multiple imputation [30] to impute smoking status (missing in 200,887 participants), diabetes (20 participants), systolic blood pressure (362 participants), and total cholesterol (17 participants) using other predictors in Model 3, mortality outcomes, and follow-up time. We repeated our analysis in several subgroups by age (< and ≥60 years), gender, and history of CVD and cancer (yes vs. no) at baseline.

For finer causes of mortality, to obtain reliable estimates, we primarily analyzed causes with ≥50 deaths and modeled the kidney measures as dichotomous variables (eGFR < vs. ≥60 ml/min/1.73m^2^ and dipstick proteinuria ≥1+ vs. none/trace). All analyses were performed using STATA version 12. All statistical tests were two-sided and statistical significance was determined as p<0.05.

## Results

The mean age of the study participants was 41.7 (SD, 10.9) years and 41.5% were women. The average eGFR was 94.2 (SD, 14.4) ml/min/1.73m^2^. Of the study participants, 1.0% (n = 3,642) had eGFR below 60 ml/min/1.73m^2^ and 3.7% (n = 13,499) had dipstick proteinuria ≥1+. Those with lower eGFR (<60 ml/min/1.73m^2^) or higher dipstick proteinuria (≥1+) were more likely to be older, male, and have diabetes, hypertension, and hypercholesterolemia compared to those with higher eGFR (≥60 ml/min/1.73m^2^) and lower proteinuria (negative/trace), respectively (Table 1).

**Table 1:** Baseline characteristics by eGFR and dipstick in Korean Heart Study, N = 367,932.

	eGFR, ml/min/1.73m^2^				Dipstick proteinuria			
	≥90	60–89	45–59	<45	None/trace	1+	2+	≥3+
	N = 227,370	N = 136,920	N = 3,136	N = 506	N = 354,433	N = 10,090	N = 2,545	N = 864
Age, years	38.2(9.3)	46.9(10.8)	61.2(9.9)	58.1(13.4)	41.6 (10.9)	42.7 (11.8)	44.2 (12.5)	47.1 (12.8)
Female	44.51	36.33	53.09	46.05	41.80	34.07	36.27	34.95
Smoking status								
Ex-smoker	5.34	8.81	14.51	12.85	14.76	14.54	16.54	22.15
Current smoker	14.45	17.00	12.34	15.22	33.39	43.72	39.72	36.97
Cardiovascular disease	0.35	0.79	3.22	3.56	0.51	1.14	1.57	1.50
Cancer	0.19	0.26	0.48	0.59	0.21	0.37	0.24	0.35
Diabetes	3.6	5.72	12.21	21.34	4.04	14.13	20.31	26.62
Anti-diabetes medication use	0.57	0.96	3.19	7.51	0.66	2.59	3.50	8.33
Fasting glucose, mg/dl	90.2(20.1)	94.3(21.8)	101.5(31.3)	103.6(35.5)	91.3 (19.6)	103.5 (38.5)	110.3 (46.3)	117.3 (54.4)
Anti-hypertension medication use	1.56	3.96	15.05	26.28	2.42	6.40	9.19	14.81
Hypertension	18.85	29.08	54.37	69.57	22.44	35.62	46.40	57.64
Systolic blood pressure, mmHg	116.9(16.0)	122.1(18.1)	134.4(22.2)	142.0(25.6)	118.8 (16.9)	124.2 (20.4)	129.0 (23.2)	137.2 (27.0)
Diastolic blood pressure, mmHg	73.9(11.3)	76.5(12.0)	78.4(13.4)	82.4(15.5)	74.8 (11.5)	78.3 (13.3)	80.7 (14.6)	82.0 (15.2)
Hypercholesterolemia	5.32	10.27	20.5	23.32	7.08	11.87	15.60	27.89
Total cholesterol, mmol/l	4.7(0.9)	5.0(0.9)	5.4(1.0)	5.3(1.4)	4.8 (0.8)	5.0 (1.0)	5.2 (1.1)	5.6 (1.3)
HDL-cholesterol, mmol/l	1.3(0.3)	1.3(0.3)	1.3(0.3)	1.2(0.3)	1.3 (0.3)	1.3 (0.3)	1.3 (0.3)	1.2 (0.3)
Triglyceride, mmol/l	1.5(1.1)	1.7(1.1)	1.9(1.2)	2.2(1.5)	1.6 (1.1)	1.8 (1.4)	2.0 (1.7)	2.3 (1.9)
LDL-cholesterol, mmol/l	2.8(0.8)	3.0(0.8)	3.3(1.0)	3.2(1.2)	2.9 (0.8)	2.9 (0.9)	3.1 (1.0)	3.4 (1.2)
Height, cm	165.3(8.3)	164.9(8.6)	160.1(8.6)	160.9(9.1)	165.1 (8.5)	165.8 (8.1)	164.9 (8.3)	164.4 (8.4)
Weight, kg	63.0(11.5)	65.3(10.8)	63.6(10.4)	62.1(10.5)	63.8 (11.2)	65.4 (12.1)	65.8 (12.9)	65.4 (12.4)
Body mass index, kg/m2	23.0(3.2)	23.9(3.0)	24.7(3.1)	23.9(3.3)	23.3 (3.1)	23.7 (3.5)	24.1 (3.8)	24.1 (3.7)
Obesity	2.12	2.65	4.91	4.55	2.26	4.05	5.89	6.25
Serum creatinine, mg/dl	0.8(0.1)	1.0(0.1)	1.2(0.2)	2.5(2.2)	0.9 (0.2)	1.0 (0.3)	1.0 (0.5)	1.3 (1.4)
eGFR, ml/min/1.73m2	103.2(8.9)	80.3(7.1)	55.7(3.5)	32.6(11.5)	94.4 (14.2)	91.3 (16.5)	86.5 (20.6)	77.4 (26.5)

**Fig 1:**
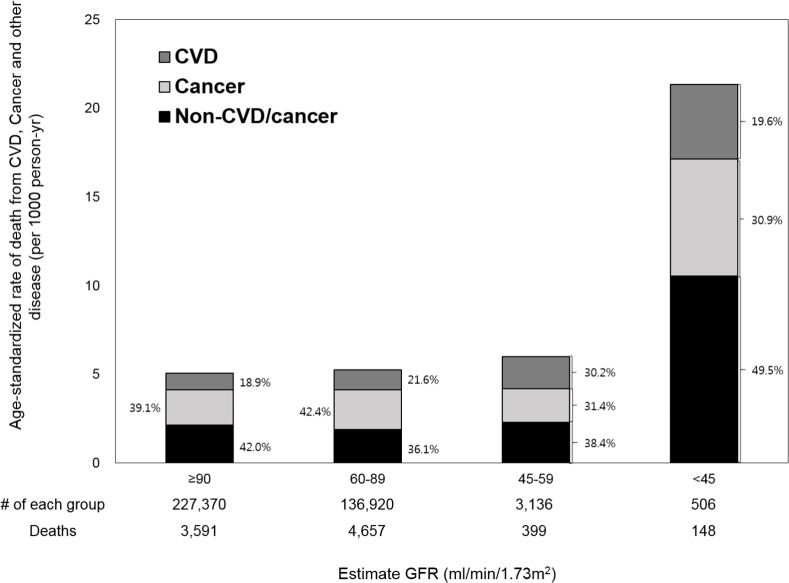
Age-standardized mortality rate (per 1000 person-years) by eGFR.

**Fig 2:**
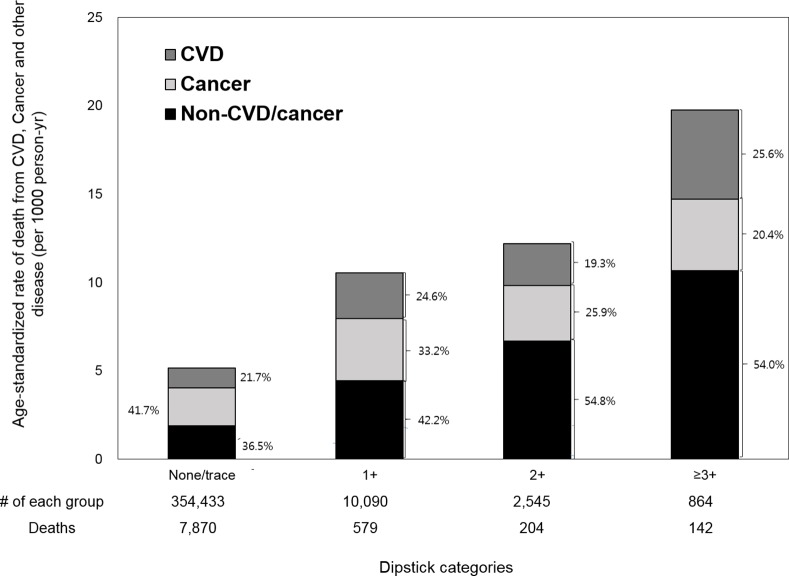
Age-standardized mortality rate (per 1000 person-years) by dipstick proteinuria.

During the median follow-up of 9.8 years, 8,795 participants died (1,608 from CVD, 4,035 from cancer, and 3,152 from other [non-CVD/non-cancer] causes). Age-standardized mortality rates were largely similar in eGFR ≥90 and 60–89 ml/min/1.73m^2^ categories but sharply increased in the eGFR <45 ml/min/1.73m^2^ category (Figs 1 and 2). Cancer was the most common cause of death only among those with eGFR 60–89 ml/min/1.73m^2^ (42.3%), but for other eGFR categories including those of eGFR <60 ml/min/1.73m^2^, non-CVD/non-cancer causes accounted for 40%-50% of cause of death (47.0% in those with eGFR <60 ml/min/1.73m^2^). Similarly, non-CVD/non-cancer mortality accounted for a great part of cause of death among those with positive proteinuria (≥1+) (54.3%), whereas cancer was the most common cause of death among those with none/trace dipstick proteinuria (41.2%). CVD was more common than cancer as a cause of death only among those with dipstick proteinuria ≥3+ (25.6% vs. 20.4%).

In crude models, there was a clear dose-response relationship between eGFR and mortality due to CVD, cancer, and non-CVD/non-cancer (Table 2). Once adjusted for demographic factor (Model 2), the associations were somewhat attenuated for all types of mortality and demonstrated U-shape. After further adjusting for potential confounders (Model 3), with eGFR 75–89 ml/min/1.73m^2^ as a reference, eGFR categories below 60 ml/min/1.73m^2^ were significantly associated with higher mortality due to CVD and non-CVD/non-cancer causes but not cancer mortality. For cancer mortality, in Model 3 we observed the lowest risk at eGFR 45–59 ml/min/1.73m^2^, when we set this category as a reference eGFR <45 ml/min/1.73m^2^ demonstrated a significant association (1.62 [1.10–2.39]). Significantly positive associations with higher eGFR categories compared to eGFR 75–89 ml/min/1.73m^2^ in Model 3 were observed for cancer and non-CVD/non-cancer mortality. We observed generally similar patterns in subgroups by gender and age (< vs. ≥60 years) (S2 and S3 Tables). Also, the results were similar among those without history of CVD or cancer at baseline (S4 Table). For dichotomous eGFR, eGFR <60 (vs. ≥60) ml/min/1.73m^2^ was significantly associated with mortality due to CVD (1.49 [1.24–1.78]) and non-CVD/non-cancer causes (1.78 [1.54–2.05]) but not mortality due to cancer (0.86 [0.73–1.01]) in Model 3.

**Table 2:** Hazard ratios (95%CI) for cause-specific mortality by eGFR in Korean Heart Study.

	eGFR, ml/min/1.73m^2^					
	≥105	90–104	75–89	60–74	45–59	<45
	N = 87,468	N = 139,902	N = 105,839	N = 31,081	N = 3,136	N = 506
CVD mortality						
Cases	103	447	570	335	118	35
Model 1	0.22 (0.18–0.27)	0.59 (0.53–0.67)	1.0	1.98 (1.73–2.27)	7.17 (5.88–8.74)	14.57 (10.35–20.49)
Model 2	1.19 (0.95–1.49)	1.05 (0.93–1.20)	1.0	1.03 (0.90–1.18)	1.62 (1.32–2.00)	3.59 (2.53–5.07)
Model 3	1.17 (0.93–1.46)	1.03 (0.91–1.17)	1.0	0.99 (0.86–1.13)	1.34 (1.90–1.66)	2.09 (1.45–3.01)
Cancer mortality						
Cases	339	1,287	1,507	741	128	33
Model 1	0.27 (0.24–0.30)	0.65 (0.60–0.70)	1.0	1.66 (1.52–1.81)	2.94 (2.45–3.52)	5.19 (3.67–7.32)
Model 2	1.28 (1.12–1.45)	1.09 (1.01–1.18)	1.0	0.91 (0.83–1.00)	0.75 (0.63–0.91)	1.46 (1.03–2.07)
Model 3	1.16 (1.02–1.32)	1.04 (0.97–1.13)	1.0	0.95 (0.87–1.04)	0.78 (0.65–0.94)	1.26 (0.88–1.80)
Non-CVD/non-cancer mortality						
Cases	396	1,019	992	512	153	80
Model 1	0.47 (0.42–0.53)	0.78 (0.71–0.85)	1.0	1.74 (1.56–1.93)	5.32 (4.49–6.31)	19.09 (15.20–23.97)
Model 2	2.01 (1.77–2.29)	1.26 (1.15–1.38)	1.0	0.99 (0.89–1.10)	1.48 (1.24–1.76)	5.86 (4.64–7.39)
Model 3	1.73 (1.51–1.91)	1.19 (1.09–1.30)	1.0	1.02 (0.92–1.14)	1.41 (1.18–1.68)	3.35 (2.61–4.31)
All-cause mortality						
Cases	838	2,753	3,069	1,588	399	148
Model 1	0.32 (0.30–0.35)	0.68 (0.64–0.71)	1.0	1.74 (1.64–1.85)	4.50 (4.05–4.90)	11.42 (9.67–13.47)
Model 2	1.53 (1.40–1.66)	1.14 (1.08–1.20)	1.0	0.96 (0.90–1.02)	1.16 (1.04–1.29)	3.24 (2.74–3.83)
Model 3	1.37 (1.26–1.49)	1.90 (1.03–1.15)	1.0	0.98 (0.92–1.04)	1.11 (1.00–1.24)	2.20 (1.84–2.63)

Model 1: unadjusted

Model 2: adjusted for age and gender

Model 3: adjusted for age, gender, total cholesterol, diabetes, cardiovascular disease, cancer, current smoker, systolic blood pressure, anti-hypertensive, body mass index and dipstick proteinuria

High proteinuria was consistently associated with CVD, cancer, and non-CVD/non-cancer mortality, with a robust dose-response relationship, even after accounting for potential confounders (Table 3). For all mortality outcomes, risk was significantly increased even in the 1+ proteinuria group. When we used data from the 13 centers (N = 178,603 and 6,448 deaths) with separate data on negative vs. trace dipstick proteinuria, the association of trace dipstick proteinuria was significant or borderline significant for mortality due to CVD (1.39 [1.15–1.69]), cancer (1.13 [0.99–1.29], p = 0.075), and other causes (1.47 [1.28–1.69]) (S5 Table). The associations were largely consistent between gender and age subgroups (S6 and S7 Tables) and among those without history of CVD or cancer (S8 Table). For dichotomous proteinuria, proteinuria (≥1+) was associated with mortality due to CVD (1.93 [1.66–2.25]), cancer (1.49 [1.32–1.68]), and other causes (2.19 [1.96–2.45]) compared to dipstick negative/trace in Model 3.

**Table 3:** Hazard ratios (95%CI) for cause-specific mortality by dipstick proteinuria in Korean Heart Study.

	Dipstick proteinuria			
	None/trace	1+	2+	≥3+
	N = 354,433	N = 10,090	N = 2,545	N = 864
CVD mortality				
Cases	1,395	135	42	36
Model 1	1.0	3.07 (2.58–3.66)	4.16 (3.06–5.65)	9.76 (7.01–13.59)
Model 2	1.0	2.40 (2.01–2.87)	2.67 (1.97–3.64)	5.33 (3.82–7.42)
Model 3	1.0	1.86 (1.55–2.24)	1.69 (1.23–2.32)	2.43 (1.68–3.51)
Cancer mortality				
Cases	3,725	219	59	32
Model 1	1.0	1.85 (1.61–2.12)	2.18 (1.69–2.82)	3.22 (2.28–4.57)
Model 2	1.0	1.49 (1.30–1.70)	1.47 (1.14–1.91)	1.86 (1.31–2.63)
Model 3	1.0	1.45 (1.26–1.66)	1.47 (1.13–1.91)	1.87 (1.30–2.68)
Non-CVD/non-cancer mortality				
Cases	2,750	225	103	74
Model 1	1.0	2.56 (2.23–2.93)	5.15 (4.23–6.27)	10.03 (7.96–12.64)
Model 2	1.0	2.13 (1.86–2.44)	3.65 (3.00–4.44)	6.12 (4.85–7.71)
Model 3	1.0	1.77 (1.54–2.03)	2.67 (2.17–3.28)	3.28 (2.52–4.28)
All-cause mortality				
Cases	7,870	579	204	142
Model 1	1.0	2.31 (2.13–2.58)	3.57 (3.11–4.10)	6.77 (5.73–7.99)
Model 2	1.0	1.88 (1.72–2.04)	2.43 (2.12–2.79)	3.94 (3.33–4.65)
Model 3	1.0	1.65 (1.51–1.80)	1.97 (1.71–2.28)	2.65 (2.20–3.18)

Model 1: unadjusted

Model 2: adjusted for age and gender

Model 3: adjusted for age, gender, total cholesterol, diabetes, cardiovascular disease, cancer, current smoker, systolic blood pressure, anti-hypertensive, body mass index and eGFR

**Fig 3:**
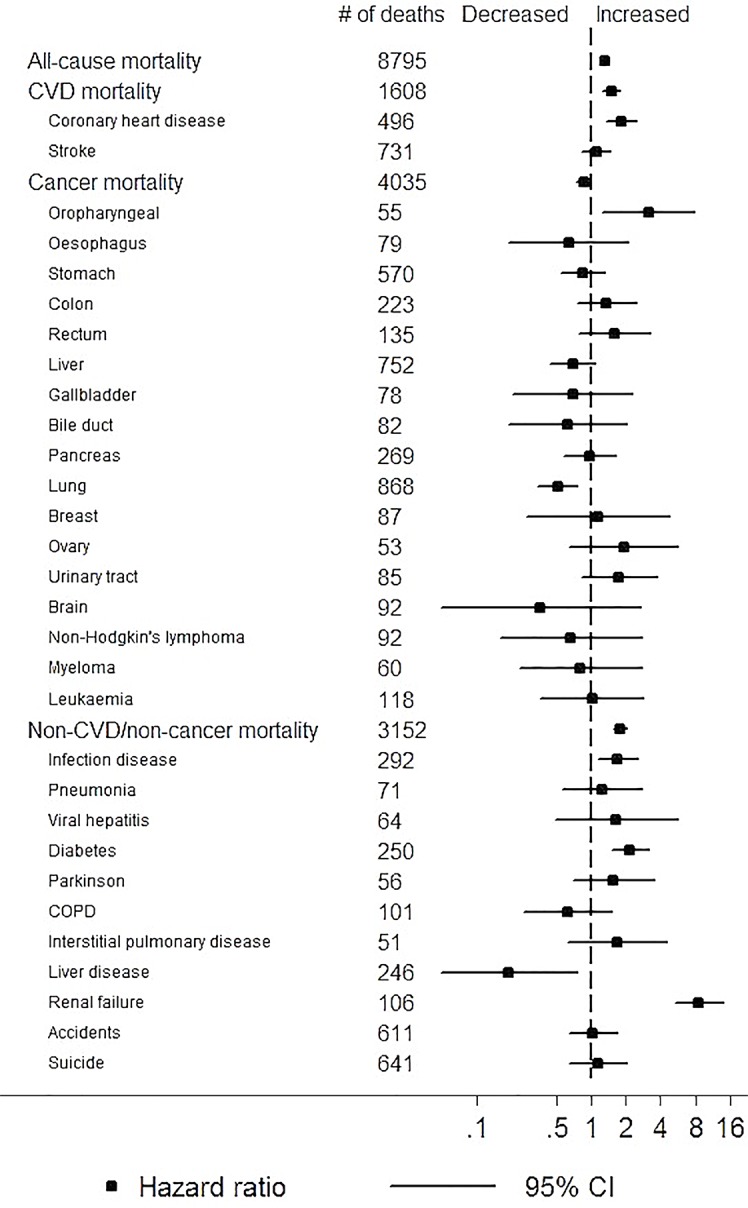
Adjusted hazard ratios of cause-specific mortality for eGFR<60 ml/min/1.73m^2^ (vs.≥60).

**Fig 4:**
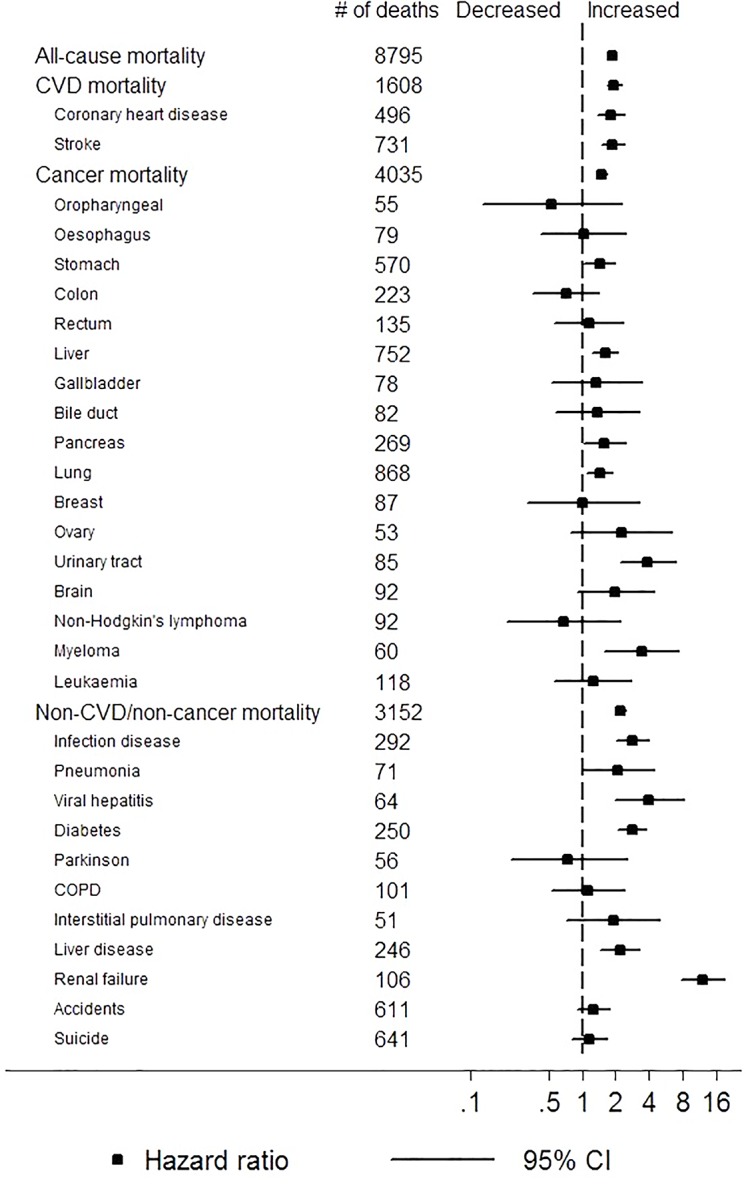
Adjusted hazard ratios of cause-specific mortality for positive dipstick proteinuria (≥+ vs. none/trace).

Examining finer mortality causes, low eGFR (<60 vs. ≥60 ml/min/1.73m^2^) and high proteinuria (≥1+ vs. none/trace) were both associated with increased risk of death from coronary disease, any infectious diseases, diabetes, and renal failure (Figs 3 and 4). In addition, low eGFR was significantly associated with increased risk of death from oropharyngeal cancer as well as paradoxically with decreased risk of death from lung cancer and liver disease (Fig 3). Proteinuria demonstrated significant associations with broader mortality causes compared to eGFR such as stroke, cancer of various organs (i.e., stomach, liver, pancreas, lung, urinary tract), myeloma, and liver disease (Fig 4). For liver outcomes, when we further adjusted for alcohol intake, liver enzymes, and seropositivity to hepatitis B and C viruses, the association remained significant for mortality from liver cancer and viral hepatitis (1.41 [1.03–1.94] and 4.06 [1.76–9.39], respectively) but not for mortality from other types of liver disease (1.38 [0.79–2.38]). Largely consistent results were observed when we excluded deaths in the first three years of follow-up (S1 and S2 Figs).

## Discussion

In this large Korean cohort study, we found that low eGFR was mainly associated with CVD and non-CVD/non-cancer mortality in adjusted analyses, whereas high proteinuria was consistently associated with each of CVD, cancer, and non-CVD/non-cancer mortality. Of note, even trace proteinuria contributed to increased mortality risk. For finer causes of death, both kidney markers were associated with increased risk of mortality due to coronary disease, infectious disease, diabetes, and renal failure. In addition, high proteinuria was also significantly associated with myriad other causes of death (i.e., stroke, cancer of stomach, liver, pancreas, lung, and urinary tract, myeloma, pneumonia, viral hepatitis, and liver disease).

As previously reported [10, 13–15], we confirmed the significant associations of eGFR and proteinuria with overall CVD mortality, independent of traditional risk factors. High proteinuria was significantly associated with death from both coronary disease and stroke, whereas low eGFR was only significantly associated with coronary disease mortality. Our finding is consistent with a few previous studies reporting a stronger association of stroke with high proteinuria than with low eGFR [31, 32]. Although the exact mechanisms for the strong association between proteinuria and stroke independently of blood pressure are uncertain, this may reflect the property of proteinuria as an indicator of systemic vascular injury and endothelial dysfunction [31].

The association of low eGFR with cancer mortality was weak and not robust in our study (only significant in unadjusted models or when we shifted the reference to eGFR 45–59 ml/min/1.73m^2^). This seems consistent with conflicting results in previous studies [17–19, 22]. A few studies reported significant associations of reduced kidney function and cancer mortality [17–19, 22], but, of note, only one study fully adjusted for important confounders like blood pressure, diabetes, lipids, and proteinuria [22]. One UK study accounted for these confounders and did not observe significant associations of CKD, defined as reduced eGFR and/or elevated proteinuria, with cancer mortality [12]. Unfortunately, this study did not report eGFR- or proteinuria-specific results. Recently, a large US cohort study with >1 million participants reported no association of low eGFR with total cancer incidence [33]. However, this study found significant associations of low eGFR with kidney and urothelial cancer incidence. In our study, although the association of low eGFR with urinary tract cancer did not reach significance, its association with kidney cancer mortality (4 cases) was borderline significant (HR 3.14 [95%CI 0.94–10.50], p-value 0.062). We also observed that low eGFR was significantly associated with high risk of oropharyngeal and low risk of lung cancer mortality. A strong U-shaped relationship between eGFR and lung cancer incidence has been shown, with the lowest incidence at eGFR 45–59 ml/min/1.73m^2^ and 1.5–2 fold higher incidence in high (≥90 ml/min/1.73m^2^) and low (<30 ml/min/1.73m^2^) eGFR ranges [33]. Lung cancer patients tend to lose weight, total body fat, and skeletal muscle mass [34], potentially explaining this U-shape, as serum creatinine correlates with muscle mass [35]. Thus our observation of the lower risk of lung cancer mortality in eGFR <60 (versus ≥60 ml/min/1.73m^2^) might reflect eGFR distribution in our study (few with eGFR <30 ml/min/1.73m^2^ and much more with higher eGFR). Nevertheless, these results for type-specific cancer in our study should be considered hypothesis-generating and validated in other settings.

To our knowledge, this is the first study reporting significant associations of proteinuria with overall and type-specific cancer mortality, although a study has reported its association with cancer incidence [36]. The associations were evident even at the level of trace or 1+ and were observed for myeloma and cancer of several gastroenterological organs (stomach, liver, and pancreas), lung, and urinary tract. The significant associations with lung and urinary tract cancer mortality are consistent with the previous study examining cancer incidence [36]. There may be several mechanisms linking proteinuria to cancer mortality. First, proteinuria may reflect prevalent cancer [37, 38]. This is particularly relevant to myeloma releasing Bence-Jones proteins, light polypeptide chains of immunoglobulins, and monoclonal gammopathy of undetermined significance in the urine. However, the significant associations in our study were consistent among those without prevalent cancer at baseline and when those who died in three years were excluded. Second, those with proteinuria may experience rapid cancer growth or spread. Indeed, endothelial dysfunction, a condition linked to proteinuria [39], is shown to contribute to invasiveness of cancer cells [40]. Finally, some investigators suggest inflammation is a mediator between proteinuria and cancer incidence since inflammation influences proliferation of cells that have lost normal growth control and promotes cancer development [36, 41].

Unlike reports from Western countries that up to half of CKD patients dies from CVD [42, 43], non-CVD/non-cancer mortality accounted for 40%-50% of cause of death than CVD or cancer mortality in our participants with CKD. This, is actually consistent with another report from East Asia [17] and may be related to the fact that the incidence and mortality rates of cardiovascular disease (particularly coronary heart disease) [44], are much lower in East Asian countries compared to Western countries [16, 44, 45]. Therefore, health care providers in East Asian countries should pay attention to non-CVD/non-cancer causes of death in the CKD care. Whether this applies to East Asians living in Western countries warrants further investigation. Regarding finer causes, both kidney measures were commonly and independently associated with overall infectious disease mortality, which is consistent with a few previous reports [19, 21, 23]. In addition, proteinuria demonstrated significant associations with death from two specific infectious diseases, pneumonia and viral hepatitis. It is known that individuals with CKD have reduced activities of the immune system [46].

Taken together, our findings suggest that persons with CKD warrant multidisciplinary care for a wide range of diseases. More specifically, in addition to CVD, special attention is needed for infectious disease prevention and management such as vaccination programs and antibiotic dosing [20, 46]. Also, our data suggest that individuals with proteinuria might merit consideration of cancer risk. Further investigations are needed regarding how to implement cancer screening in the context of CKD. Nevertheless, the assessment of proteinuria is already recommended in some clinical populations such as diabetes and hypertension. Simultaneously, cancer screening programs are developed in several countries/regions for some cancers such as lung cancer (aged 55–80 years with some smoking history) [47] and stomach cancer (aged ≥40 years) [48]. Thus, it seems reasonable to encourage persons with proteinuria to strictly adhere to those cancer screening programs.

This study has several limitations. First, our study participants were in routine health assessments at private health promotion centers and thus may not necessarily represent the general population in Korea, particularly regarding socioeconomic status and age distribution [24]. Second, our findings may not be generalizable to those with advanced CKD (e.g., on dialysis) since few participants had severely reduced eGFR. Third, cystatin C was not available. Cystatin C-based eGFR is a stronger predictor of mortality and CVD outcomes than creatinine-based eGFR [49, 50]. Thus, investigations with cystatin C for cancer and non-CVD/non-cancer mortality are warranted. Fourth, we used a semi-quantitative urinary dipstick test as a measure of kidney damage. As it does not account for urine concentration, it is likely that there were some misclassifications of proteinuria. However, this kind of misclassification usually biases the association toward null. Finally, the outcome definition was based on ICD-10 codes from death certificates (S8 Table). There have been debates about the accuracy of this approach[51], and the approach of coding primary cause may not be uniform across physicians particularly when several comorbidities are present, e.g., diabetes and its complications. Nonetheless, it is widely used in literature [52–54] and the accuracy of cancer death codes is high [55, 56].

In conclusion, low eGFR was mainly associated with mortality due to CVD and non-CVD/non-cancer causes, whereas proteinuria was consistently associated mortality due to CVD, cancer, and other causes. Our study provides further evidence that persons with CKD (particularly those with proteinuria) are at high risk for not only CVD but also other causes of deaths such as cancer and infectious disease, suggesting that they may benefit from multidisciplinary prevention and management strategies.

## Supporting Information

S1 Fig Adjusted hazard ratios of cause-specific mortality for eGFR<60 ml/min/1.73m^2^ (vs. ≥60), excluding those who died in 3 years. (PDF)

S2 Fig Adjusted hazard ratios of cause-specific mortality for positive dipstick proteinuria (≥+ vs. none/trace), excluding those who died in 3 years. (PDF)

S1 Table ICD-10 codes of cause-specific mortality. (DOCX)

S2 Table Hazard ratios (95%CI)* for cause-specific mortality by eGFR and age. (DOCX)

S3 Table Hazard ratios (95%CI)* for cause-specific mortality by eGFR and gender. (DOCX)

S4 Table Hazard ratios (95%CI)* for cause-specific mortality by eGFR and prevalent CVD or cancer in Korean Heart Study. (DOCX)

S5 Table Hazard ratios (95%CI) for cause-specific mortality by dipstick proteinuria in Korean Heart Study (excluding hospital #7), N = 178,603. (DOCX)

S6 Table Hazard ratios (95%CI)* for cause-specific mortality by dipstick and age. (DOCX)

S7 Table Hazard ratios (95%CI)* for cause-specific mortality by dipstick and gender. (DOCX)

S8 Table Hazard ratios (95%CI)* for cause-specific mortality by dipstick proteinuria in Korean Heart Study. (DOCX)
