# Supplementary figures and images for: Association of Kidney Disease Measures with Cause-Specific Mortality: The Korean Heart Study
Source: PLoS One. 2016 Apr 19;11(4):e0153429. doi: 10.1371/journal.pone.0153429 (PMC4836674; doi:10.1371/journal.pone.0153429)

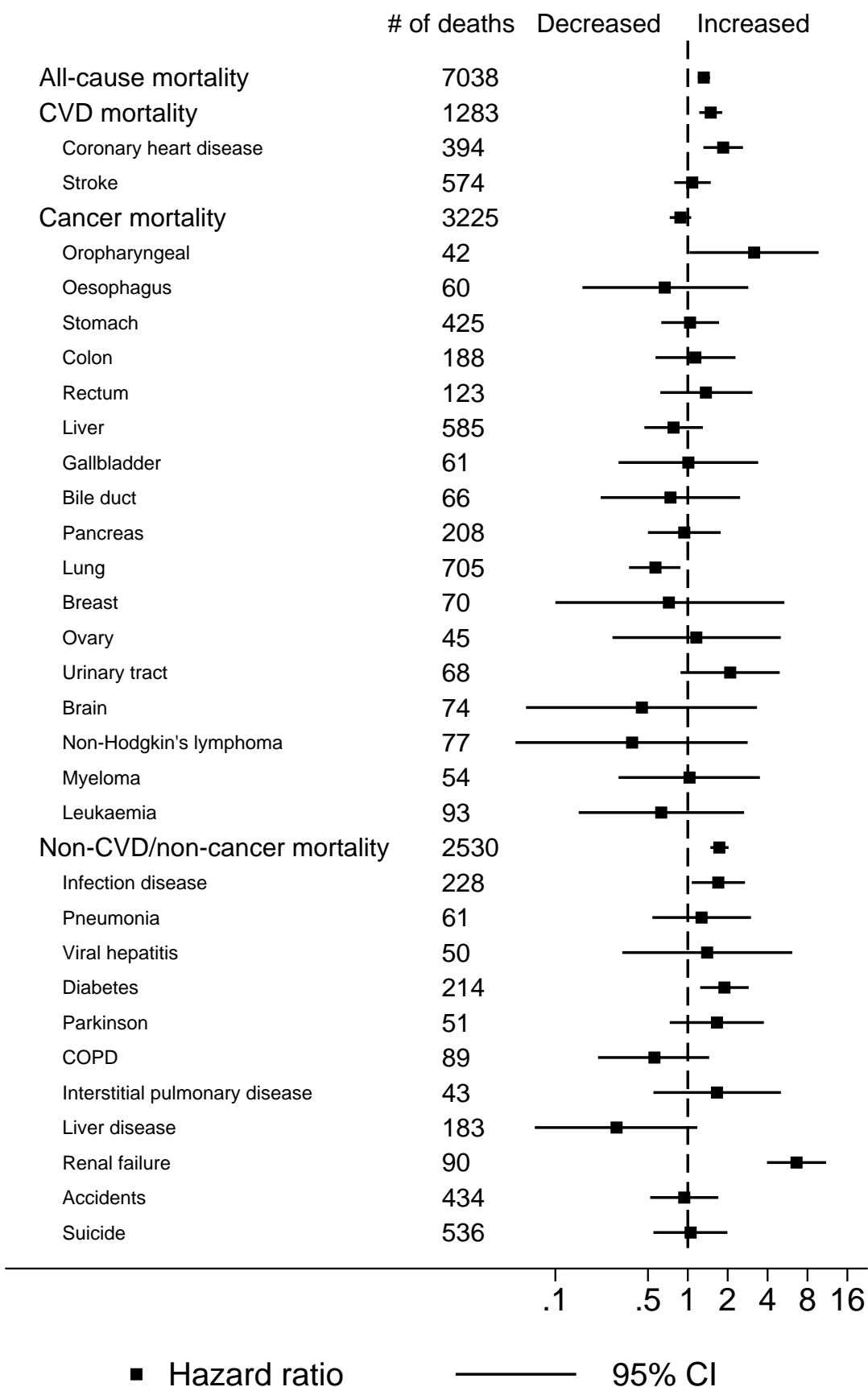

Supplement: S1 Fig — (PDF) [file pone.0153429.s001.pdf]

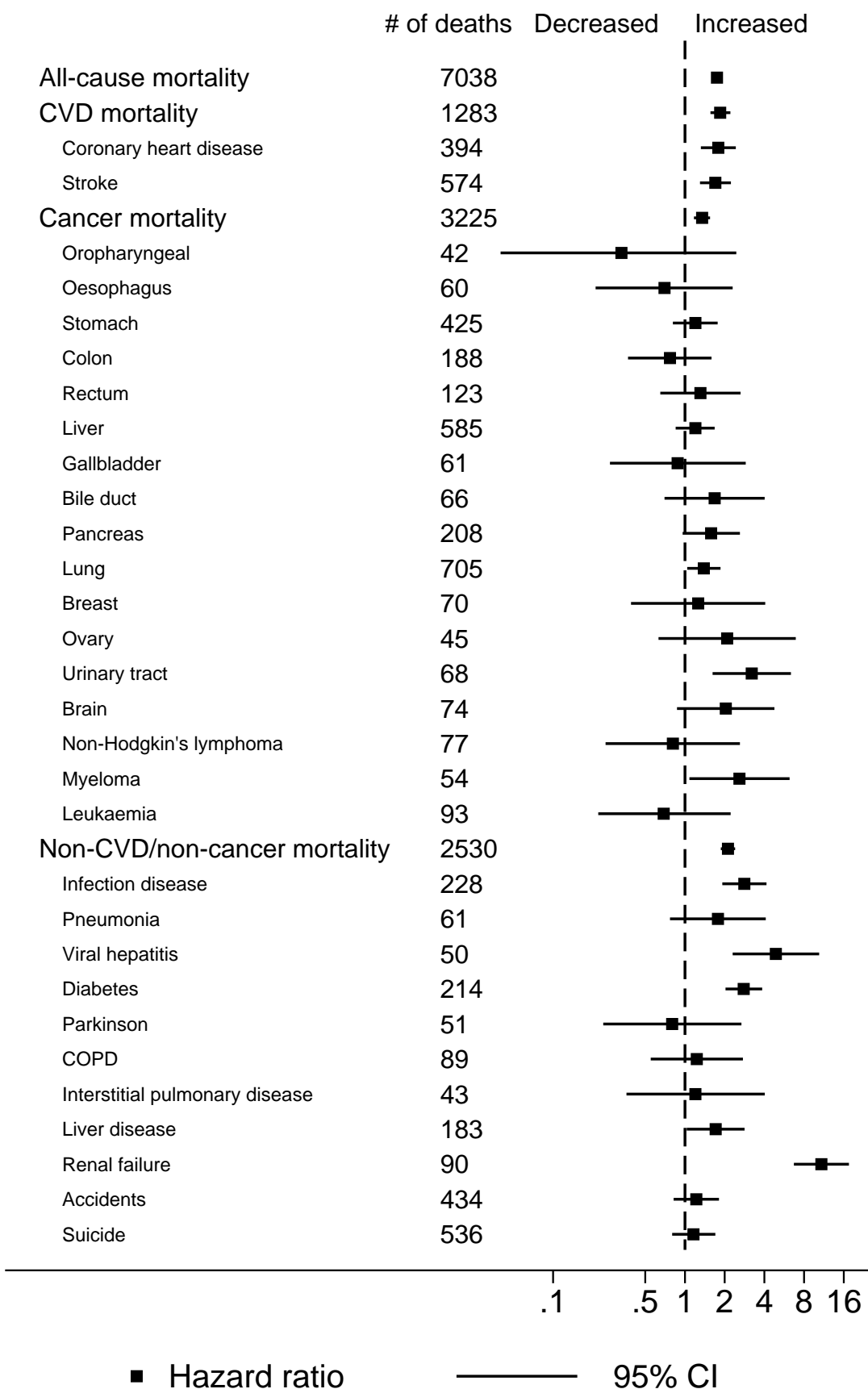

Supplement: S2 Fig — (PDF) [file pone.0153429.s002.pdf]
