# Supplementary material for: Association of Kidney Disease Measures with Cause-Specific Mortality: The Korean Heart Study
Source: PLoS One. 2016 Apr 19;11(4):e0153429. doi: 10.1371/journal.pone.0153429 (PMC4836674; doi:10.1371/journal.pone.0153429)
Supplement: S1 Table — (DOCX) [file pone.0153429.s003.docx]

**S1 Table.** ICD-10 codes of cause-specific mortality

| **Cause-specific mortality** | **ICD-10 codes** |
| --- | --- |
| **CVD mortality** |  |
| Coronary heart disease | I20-I25 |
| Stroke | I60-I69 |
| **Cancer mortality** |  |
| Oropharyngeal | C00-C14 |
| Oesophagus | C15 |
| Stomach | C16 |
| Colon | C18 |
| Rectum | C19-C20 |
| Liver | C22 |
| Gallbladder | C23 |
| Bile duct | C24 |
| Pancreas | C25 |
| Lung | C34 |
| Breast | C50 |
| Ovary | C56 |
| Urinary tract |  |
| Brain | C71 |
| Non-Hodgkin’s lymphoma | C82-C85 |
| Myeloma | C90 |
| Leukaemia | C91-C95 |
| **Non-CVD/non-cancer mortality** |  |
| Infection disease | A00 -B99, G00-G04, G05, G08, I30, I32-I33, I38-I41, I80, I88, J00-J22, J31-J32, J35, J37, J40-J42, J85-J86, K05, K29, K65, K67, K73, K80-K81, L02-L05, L08, M00-M01, M03 |
| Pneumonia | J12-J18 |
| Viral hepatitis | B15-B19 |
| Diabetes | E10-E14 |
| Parkinson | G20-G21 |
| COPD | J42-J44 |
| Interstitial pulmonary disease | J80-J84 |
| Liver disease | K70-K77 |
| Renal failure | N17-N19 |
| Accidents | V00-V95, W00-W95, X00-X59 |
| Suicide | X60-X84 |
