# Supplementary material for: Association of Kidney Disease Measures with Cause-Specific Mortality: The Korean Heart Study
Source: PLoS One. 2016 Apr 19;11(4):e0153429. doi: 10.1371/journal.pone.0153429 (PMC4836674; doi:10.1371/journal.pone.0153429)
Supplement: S2 Table — (DOCX) [file pone.0153429.s004.docx]

**S2 Table**. Hazard ratios (95%CI)* for cause-specific mortality by eGFR and age

|  | eGFR, ml/min/1.73m^2^ | | | | | |
| --- | --- | --- | --- | --- | --- | --- |
|  | ≥105 | 90-104 | 75-89 | 60-74 | 45-59 | <45 |
| **N (age<60/age>=60)** | 87,358/110 | 133,768/6,134 | 94,769/11,070 | 22,406/8,675 | 1,310/1,826 | 244/262 |
| CVD mortality | 101/2 | 300/147 | 291/279 | 100/235 | 16/102 | 7/28 |
| Age<60 | 1.09 (0.86-1.39) | 0.99 (0.84-1.17) | 1.0 | 0.96 (0.77-1.21) | 1.23 (0.73-2.07) | 1.72 (0.76-3.89) |
| Age>=60 | 0.77 (0.19-3.10) | 1.14 (0.93-1.39) | 1.0 | 0.99 (0.83-1.18) | 1.35 (1.06-1.71) | 2.20 (1.45-3.33) |
| Cancer mortality | 334/5 | 962/325 | 874/633 | 273/468 | 24/104 | 9/24 |
| Age<60 | 1.26 (1.10-1.45) | 1.05 (0.96-1.15) | 1.0 | 0.95 (0.83-1.09) | 0.89 (0.59-1.35) | 1.99 (1.00-3.96) |
| Age>=60 | 0.81 (0.33-1.95) | 1.02 (0.89-1.17) | 1.0 | 0.99 (0.88-1.12) | 0.88 (0.71-1.09) | 1.38 (0.91-2.11) |
| Non-CVD/non-cancer mortality | 391/5 | 778/241 | 534/458 | 165/347 | 17/136 | 24/56 |
| Age<60 | 1.67 (1.45-1.93) | 1.27 (1.13-1.42) | 1.0 | 0.99 (0.83-1.19) | 1.08 (0.66-1.76) | 4.72 (2.98-7.49) |
| Age>=60 | 0.82 (0.34-2.01) | 1.08 (0.92-1.27) | 1.0 | 0.98 (0.85-1.13) | 1.27 (1.04-1.56) | 2.65 (1.96-3.59) |
| All-cause mortality | 826/12 | 2,040/713 | 1,699/  1,370 | 538/1,050 | 57/342 | 40/108 |
| Age<60 | 1.38 (1.26-1.52) | 1.11 (1.04-1.08) | 1.0 | 0.97 (0.88-1.07) | 1.05 (0.80-1.37) | 2.96 (2.10-4.16) |
| Age>=60 | 0.82 (0.46-1.46) | 1.07 (0.97-1.17) | 1.0 | 0.98 (0.91-1.07) | 1.13 (1.00-1.28) | 2.10 (1.70-2.60) |

* adjusted for age, total cholesterol, diabetes, cardiovascular disease, cancer, current smoker, systolic blood pressure, anti-hypertensive, body mass index and dipstick proteinuria
