# Supplementary material for: Association of Kidney Disease Measures with Cause-Specific Mortality: The Korean Heart Study
Source: PLoS One. 2016 Apr 19;11(4):e0153429. doi: 10.1371/journal.pone.0153429 (PMC4836674; doi:10.1371/journal.pone.0153429)
Supplement: S3 Table — (DOCX) [file pone.0153429.s005.docx]

**S3 Table**. Hazard ratios (95%CI)* for cause-specific mortality by eGFR and gender

|  | eGFR, ml/min/1.73m^2^ | | | | | |
| --- | --- | --- | --- | --- | --- | --- |
|  | ≥105 | 90-104 | 75-89 | 60-74 | 45-59 | <45 |
| **N (men/women)** | 41,958/45,510 | 84,219/55,683 | 68,605/37,234 | 18,577/12,504 | 1,471/1,665 | 273/233 |
| CVD mortality | 76/27 | 322/125 | 413/157 | 222/113 | 72/46 | 20/15 |
| Men | 1.12 (0.86-1.45) | 1.01 (0.87-1.18) | 1.0 | 1.01 (0.85-1.19) | 1.36 (1.04-1.78) | 2.08 (1.29-3.38) |
| Women | 1.38 (0.88-2.17) | 1.10 (0.86-1.40) | 1.0 | 0.95 (0.75-1.22) | 1.28 (0.91-1.81) | 1.19 (0.67-2.11) |
| Cancer mortality | 214/125 | 939/348 | 1,167/340 | 535/206 | 81/47 | 25/8 |
| Men | 1.15 (0.98-1.34) | 1.03 (0.94-1.12) | 1.0 | 0.91 (0.82-1.02) | 0.66 (0.52-0.83) | 1.27 (0.84-1.92) |
| Women | 1.07 (0.85-1.35) | 1.09 (0.93-1.27) | 1.0 | 1.07 (0.90-1.27) | 1.13 (0.82-1.54) | 1.28 (0.62-2.64) |
| Non-CVD/non-cancer mortality | 261/135 | 752/267 | 727/265 | 359/153 | 102/51 | 51/29 |
| Men | 1.77 (1.52-2.07) | 1.22 (1.10-1.36) | 1.0 | 1.04 (0.92-1.19) | 1.44 (1.16-1.79) | 3.29 (2.42-4.49) |
| Women | 1.58 (1.25-2.03) | 1.09 (0.91-1.29) | 1.0 | 0.97 (0.79-1.18) | 1.32 (0.96-1.80) | 3.21 (2.09-4.93) |
| All-cause mortality | 551/287 | 2,013/740 | 2,307/762 | 1,116/472 | 255/144 | 96/52 |
| Men | 1.36 (1.23-1.51) | 1.09 (1.02-1.16) | 1.0 | 0.97 (0.90-1.05) | 1.04 (0.91-1.19) | 2.12 (1.71-2.64) |
| Women | 1.33 (1.14-1.55) | 1.09 (0.98-1.21) | 1.0 | 1.01 (0.90-1.14) | 1.27 (1.06-1.53) | 2.33 (1.72-3.16) |

***** adjusted for age, total cholesterol, diabetes, cardiovascular disease, cancer, current smoker, systolic blood pressure, anti-hypertensive, body mass index and dipstick proteinuria
