# Supplementary material for: Association of Kidney Disease Measures with Cause-Specific Mortality: The Korean Heart Study
Source: PLoS One. 2016 Apr 19;11(4):e0153429. doi: 10.1371/journal.pone.0153429 (PMC4836674; doi:10.1371/journal.pone.0153429)
Supplement: S4 Table — (DOCX) [file pone.0153429.s006.docx]

**S4 Table**. Hazard ratios (95%CI)* for cause-specific mortality by eGFR and prevalent CVD or cancer in Korean Heart Study

|  | eGFR, ml/min/1.73m^2^ | | | | | | |
| --- | --- | --- | --- | --- | --- | --- | --- |
|  | ≥105 | 90-104 | 75-89 | 60-74 | 45-59 | 30-44 | <30 |
| **N (without/with CVD)** | 87,266/202 | 139,316/586 | 105,157/682 | 30,684/397 | 3,035/101 | 327/11 | 161/7 |
| CVD mortality | 102/1 | 436/11 | 552/18 | 319/16 | 108/10 | 21/2 | 11/1 |
| Without history of CVD at baseline | 1.18 (0.94-1.48) | 1.03 (0.91-1.18) | 1.0 | 0.99 (0.86-1.14) | 1.33 (1.07-1.66) | 1.83 (1.16-2.87) | 2.37 (1.25-4.51) |
| With history of CVD at baseline | 0.82 (0.10-6.70) | 1.04 (0.48-2.25) | 1.0 | 1.02 (0.51-2.06) | 1.61 (0.68-3.78) | 2.30 (0.48-11.02) | 6.80 (0.77-59.86) |
| **N (without/with cancer)** | 87,327/141 | 139,620/282 | 105,579/260 | 30,983/98 | 3,121/15 | 335/3 | 168/0 |
| Cancer mortality | 334/5 | 1,276/11 | 1,493  /14 | 733/8 | 127/1 | 26/0 | 7/0 |
| Without history of cancer at baseline | 1.15 (1.01-1.31) | 1.05 (0.97-1.13) | 1.0 | 0.94 (0.86-1.03) | 0.78 (0.64-0.94) | 1.28 (0.86-1.91) | 1.17 (0.55-2.52) |
| With history of cancerat baseline | 1.69 (0.52-5.45) | 1.00 (0.44-2.29) | 1.0 | 1.72 (0.69-4.25) | 0.68 (0.09-5.39) | 0.00 (0.00-0.00) | 0.00 (0.00-0.00) |

* adjusted for age, gender, total cholesterol, diabetes, cardiovascular disease, cancer, current smoker, systolic blood pressure, anti-hypertensive, body mass index and dipstick proteinuria
