# Supplementary material for: Association of Kidney Disease Measures with Cause-Specific Mortality: The Korean Heart Study
Source: PLoS One. 2016 Apr 19;11(4):e0153429. doi: 10.1371/journal.pone.0153429 (PMC4836674; doi:10.1371/journal.pone.0153429)
Supplement: S5 Table — (DOCX) [file pone.0153429.s007.docx]

**S5 Table**. Hazard ratios (95%CI) for cause-specific mortality by dipstick proteinuria in Korean Heart Study (excluding hospital #7), N=178,603

|  | Dipstick proteinuria | | | | |
| --- | --- | --- | --- | --- | --- |
|  | none | trace | + | ++ | ≥+++ |
|  | N=154,739 | N=14,272 | N=7,320 | N=1,598 | N=674 |
| CVD mortality |  |  |  |  |  |
| Case | 910 | 117 | 113 | 33 | 32 |
| Model 1 | 1.0 | 1.87 (1.55-2.26) | 3.19 (2.68-3.82) | 4.33 (3.18-5.89) | 10.17 (7.30-14.17) |
| Model 2 | 1.0 | 1.57 (1.29-1.90) | 2.35 (1.93-2.86) | 2.81 (1.98-3.98) | 5.59 (3.92-7.95) |
| Model 3 | 1.0 | 1.39 (1.15-1.69) | 1.88 (1.53-2.30) | 1.82 (1.27-2.60) | 2.61 (1.75-3.88) |
| Cancer mortality |  |  |  |  |  |
| Case | 2,437 | 245 | 187 | 47 | 29 |
| Model 1 | 1.0 | 1.46 (1.28-1.68) | 1.61 (1.47-1.77) | 1.98 (1.60-2.45) | 3.31 (2.51-4.36) |
| Model 2 | 1.0 | 1.16 (1.02-1.32) | 1.44 (1.24-1.67) | 1.52 (1.14-2.03) | 1.92 (1.33-2.76) |
| Model 3 | 1.0 | 1.13 (0.99-1.29) | 1.40 (1.20-1.63) | 1.53 (1.14-2.05) | 1.89 (1.28-2.77) |
| Non-CVD/non-cancer mortality |  |  |  |  |  |
| Case | 1,733 | 232 | 195 | 79 | 59 |
| Model 1 | 1.0 | 1.85 (1.62-2.12) | 2.67 (2.33-3.06) | 5.36 (4.40-6.53) | 10.47 (8.30-13.19) |
| Model 2 | 1.0 | 1.58 (1.37-1.81) | 2.17 (1.87-2.52) | 3.68 (2.94-4.62) | 5.65 (4.35-7.32) |
| Model 3 | 1.0 | 1.47 (1.28-1.69) | 1.84 (1.58-2.14) | 2.87 (2.27-3.62) | 3.06 (2.27-4.13) |
| All-cause mortality |  |  |  |  |  |
| Case | 5,080 | 594 | 495 | 159 | 120 |
| Model 1 | 1.0 | 1.30 (1.19-1.42) | 2.08 (1.90-2.28) | 3.20 (2.73-3.75) | 5.61 (4.68-6.72) |
| Model 2 | 1.0 | 1.37 (1.26-1.49) | 1.85 (1.69-2.03) | 2.48 (2.12-2.91) | 3.83 (3.20-4.59) |
| Model 3 | 1.0 | 1.29 (1.19-1.41) | 1.65 (1.50-1.81) | 2.08 (1.76-2.44) | 2.57 (2.10-3.14) |

Model 1: crude

Model 2: adjusted for age and gender

Model 3: adjusted for age, gender, total cholesterol, diabetes, cardiovascular disease, cancer, current smoker, systolic blood pressure, anti-hypertensive, body mass index and eGFR
