# Supplementary material for: Association of Kidney Disease Measures with Cause-Specific Mortality: The Korean Heart Study
Source: PLoS One. 2016 Apr 19;11(4):e0153429. doi: 10.1371/journal.pone.0153429 (PMC4836674; doi:10.1371/journal.pone.0153429)
Supplement: S6 Table — (DOCX) [file pone.0153429.s008.docx]

**S6 Table**. Hazard ratios (95%CI)* for cause-specific mortality by dipstick and age

|  | Dipstick proteinuria | | | |
| --- | --- | --- | --- | --- |
|  | None/trace | 1+ | 2+ | ≥3+ |
| **N (age<60/age>=60)** | 327,891/26,542 | 9,070/1,020 | 2,197/348 | 697/167 |
| CVD mortality | 698/697 | 73/62 | 22/20 | 22/14 |
| Age<60 | 1.0 | 2.19 (1.70-2.80) | 2.31 (1.50-3.57) | 4.03 (2.48-6.55) |
| Age>=60 | 1.0 | 1.54 (1.18-2.02) | 1.32 (0.83-2.08) | 1.67 (0.96-2.92) |
| Cancer mortality | 2,301/1,424 | 125/94 | 31/28 | 19/13 |
| Age<60 | 1.0 | 1.52 (1.26-1.82) | 1.58 (1.10-2.27) | 2.38 (1.48-3.84) |
| Age>=60 | 1.0 | 1.36 (1.10-1.68) | 1.31 (0.89-1.92) | 1.28 (0.73-2.23) |
| Non-CVD/non-cancer mortality | 1,696/1,054 | 123/102 | 53/50 | 37/37 |
| Age<60 | 1.0 | 1.83 (1.52-2.21) | 3.03 (2.28-4.02) | 4.07 (2.81-5.90) |
| Age>=60 | 1.0 | 1.63 (1.32-2.01) | 2.40 (1.78-3.23) | 3.42 (2.40-4.87) |
| All-cause mortality | 4,695/3,175 | 321/258 | 106/98 | 78/64 |
| Age<60 | 1.0 | 1.76 (1.57-1.97) | 2.29 (1.88-2.79) | 3.55 (2.77-4.55) |
| Age>=60 | 1.0 | 1.50 (1.32-1.71) | 1.71 (1.38-2.10) | 2.18 (1.68-2.82) |

* adjusted for age, gender, total cholesterol, diabetes, cardiovascular disease, cancer, current smoker, systolic blood pressure, anti-hypertensive, body mass index and eGFR
