# Supplementary material for: Association of Kidney Disease Measures with Cause-Specific Mortality: The Korean Heart Study
Source: PLoS One. 2016 Apr 19;11(4):e0153429. doi: 10.1371/journal.pone.0153429 (PMC4836674; doi:10.1371/journal.pone.0153429)
Supplement: S7 Table — (DOCX) [file pone.0153429.s009.docx]

**S7 Table**. Hazard ratios (95%CI)* for cause-specific mortality by dipstick and gender

|  | Dipstick proteinuria | | | |
| --- | --- | --- | --- | --- |
|  | None/trace | 1+ | 2+ | ≥3+ |
| **N (men/women)** | 206,267/148,166 | 6,652/3,438 | 1,622/923 | 562/302 |
| CVD mortality | 955/440 | 112/23 | 32/10 | 26/10 |
| Men | 1.0 | 2.00 (1.63-2.45) | 1.82 (1.26-2.61) | 2.28 (1.49-3.51) |
| Women | 1.0 | 1.52 (0.99-2.32) | 1.47 (0.77-2.81) | 3.80 (1.94-7.43) |
| Cancer mortality | 2,708/1,017 | 179/40 | 47/12 | 27/5 |
| Men | 1.0 | 1.41 (1.21-1.65) | 1.42 (1.06-1.91) | 1.84 (1.24-2.72) |
| Women | 1.0 | 1.49 (1.08-2.05) | 1.51 (0.85-2.69) | 1.64 (0.67-4.00) |
| Non-CVD/non-cancer mortality | 1,946/804 | 174/51 | 78/25 | 54/20 |
| Men | 1.0 | 1.71 (1.45-2.00) | 2.70 (2.13-3.41) | 3.35 (2.49-4.50) |
| Women | 1.0 | 2.07 (1.55-2.75) | 2.83 (1.86-4.30) | 4.72 (2.91-7.67) |
| All-cause mortality | 5,609/2,261 | 465/114 | 157/47 | 107/35 |
| Men | 1.0 | 1.63 (1.48-1.80) | 1.97 (1.68-2.32) | 2.64 (2.15-3.23) |
| Women | 1.0 | 1.71 (1.41-2.07) | 2.04 (1.51-2.75) | 3.46 (2.43-4.93) |

* adjusted for age, total cholesterol, diabetes, cardiovascular disease, cancer, current smoker, systolic blood pressure, anti-hypertensive, body mass index and eGFR
