# Supplementary material for: Association of Kidney Disease Measures with Cause-Specific Mortality: The Korean Heart Study
Source: PLoS One. 2016 Apr 19;11(4):e0153429. doi: 10.1371/journal.pone.0153429 (PMC4836674; doi:10.1371/journal.pone.0153429)
Supplement: S8 Table — (DOCX) [file pone.0153429.s010.docx]

**S8 Table**. Hazard ratios (95%CI)* for cause-specific mortality by dipstick proteinuria in Korean Heart Study

|  | Dipstick proteinuria | | | |
| --- | --- | --- | --- | --- |
|  | None/trace | 1+ | 2+ | ≥3+ |
| **N (without/with CVD)** | 352,615/1,818 | 9,975/115 | 2,505/40 | 851/13 |
| CVD mortality | 1,349/46 | 125/10 | 39/3 | 36/0 |
| Without CVD | 1.0 | 1.81 (1.50-2.18) | 1.73 (1.24-2.40) | 2.48 (1.71-3.58) |
| With CVD | 1.0 | 3.22 (1.56-6.68) | 1.60 (0.46-5.61) | 0.00 (0.00-0.00) |
| **N (without/with cancer)** | 353,680/753 | 10,053/37 | 2,539/6 | 861/3 |
| Cancer mortality | 3,689/36 | 217/2 | 59/0 | 31/1 |
| Without cancer | 1.0 | 1.46 (1.27-1.68) | 1.47 (1.13-1.91) | 1.84 (1.28-2.66) |
| With cancer | 1.0 | 1.33 (0.29-5.97) | 0.00 (0.00-0.00) | 8.24 (0.66-102.07) |

* adjusted for age, gender, total cholesterol, diabetes, cardiovascular disease, cancer, current smoker, systolic blood pressure, anti-hypertensive, body mass index and eGFR
